# LoPATraN: Low Power Asset Tracking by Means of Narrow Band IoT (NB-IoT) Technology

**DOI:** 10.3390/s21113772

**Published:** 2021-05-29

**Authors:** Stefano Parrino, Giacomo Peruzzi, Alessandro Pozzebon

**Affiliations:** Department of Information Engineering and Mathematics, University of Siena, 53100 Siena, Italy; parrino2@unisi.it (S.P.); peruzzi@diism.unisi.it (G.P.)

**Keywords:** NB-IoT, GPS, tracking system, asset tracking, LPWAN, IoT, low power devices

## Abstract

The narrowband Internet-of-Things (NB-IoT) communication standard is gaining momentum within the big picture of the Internet-of-Things (IoT) owing to its capabilities of ensuring pervasive and wide coverage while limiting power consumption. Therefore, it turns out to be a valuable enabling technology within a considerable number of applications. Apart from traditional remote monitoring and data acquisition purposes where comparable Low Power Wide Area Network (LPWAN) facilities have ruled for years, NB-IoT can potentially carve out space within specific alcoves in which low latency, low power, high data-rates and ubiquitous coverage are fundamentals requirements. Long term asset tracking definitely falls within such niches, and in particular NB-IoT can become a valuable alternative to be exploited by both replacing the conventional Global Position System (GPS) system, or supporting it. To this end, this paper proposes an innovative tracking system prototype for asset shipping which relies on two enabling technologies: GPS and NB-IoT. While position transmission is always put into effect via NB-IoT, it can be fetched by resorting to both GPS (like a standard tracker) or NB-IoT (thus establishing a GPS-less method). As a result, two localization techniques are arranged: the former one is preciser but energy hungrier, while the latter one is coarser but more low power. Such working principles were successfully tested on the field by means of two road tests in as much itineraries. Tests results are in agreement with the expectations underlying the two working principles since the finer one provides a more accurate tracking. In addition, a consumption analysis was also performed aiming at assessing the prototype lifetime. Finally, tests pursuing the assessment of the tracking error were carried out underling the fact that it strongly depends on the geographic deployment of NB-IoT towers.

## 1. Introduction

Supply chain management has undergone a never ceasing surge throughout the decades. Specifically, shipping companies have pursued and fostered the development of systems able to realise remote long term tracking and monitoring of goods so to provide a beneficial support to all of the decision phases such companies have to face and issue. A way to put into effect the aforementioned systems could be to provide them with connectivity, which has to satisfy the essential requirements of wide coverage and low power consumption: the former ensures the remote monitoring, while the latter guarantees the long term one. Therefore, an optimal trade-off is to resort to the robustness and reliability of Low Power Wide Area Network (LPWAN) technologies which let Internet of Things (IoT) devices to run for years only relying on sole batteries.

From containers to parcels, from perishables to storage products, from wastes to brand new items: assets can be taken into account in a broad sense, and their monitoring during their shipment can be crucial for a plethora of reasons. These activities may be carried out by equipping assets with wireless sensor nodes communicating via LPWANs, because they can broadcast assets position to a remote server so to accomplish tracking and supervision. In so doing, reliable and secure shipment can be done thus enhancing the whole production and supply chain.

Initially, pen and paper was the only strategy to put into effect asset tracking. Then, spreadsheets or bar-codes were employed. However, these are rudimentary and meagre tracking systems which are often devoid of automatism thus resulting to be quite error prone. Subsequently, and for thirty years now, Global Positioning System (GPS) has started to be a standard concerning localisation purposes, and thus it has been widely employed since then and on. The associated drawback, though, is that GPS modules are way too power hungry thus being not so appropriate for long term tracking whenever the only source of power the tracker relies on is non renewable. Providing that the tracker exploits LPWAN technologies to send its location, a viable alternative is the one of resorting to trilateration algorithms to be applied on Receiver Signal Strength Indicators (RSSIs), Time of Arrival (ToA), Time Difference Of Arrival (TDoA) or Angle of Arrival (AoA) supposing multiple receivers are spread-out. For instance, this strategy can be harnessed for Long Range Wide Area Network (LoRaWAN) transceivers as long as a pervasive deployment of LoRaWAN gateway is ensured: unfortunately, this is nearly unfeasible when such coverage has to reach extensive areas as the one to which assets could be subject during their shipment. On the other hand, cellular technologies have already many receivers distributed worldwide. However, such standards usually require a massive amount of energy, with respect to LPWANs, to run. But, the newly issued Narrowband Internet of Things (NB-IoT) standard is a promising technology ensuring wide and pervasive coverage while limiting power consumption. Therefore, it perfectly suits as a solution to the remote long term tracking and monitoring problem.

In this paper, an innovative tracking system for asset shipping is presented. In particular, its architecture, its working principle along with the whole system description are shown. It is mainly composed of a prototype of a tracker, a back-end server and a web application so that users may check tracker position in real time. The tracker prototype simultaneously makes use of two enabling technologies: GPS and NB-IoT. Respectively, the former for tracking, while the latter both for tracking and communication. Indeed, tracker position may be retrieved by following two approaches (i.e., coarse but low power, or fine but energy requiring) and then remotely sent via NB-IoT by means of the Message Queue Telemetry Transport (MQTT) protocol. The rougher localisation method is put into effect by fetching the position of the Long Term Evolution (LTE) base stations, named as evolved Node B (eNodeB), to which the tracker pairs to while it synchronises to NB-IoT network. The more precise localization method is based on the use of GPS. Of course, with the view of limit consumption, GPS position is sporadically requested, and no trilateration is locally made whenever the eNodeB position is exploited.

The rest of the paper is arranged as follows. Section 2 gathers a literature review made of some related works, while Section 3 shows the system architecture and the working principles the tracker prototype executes. Section 4 describes hardware setup of the tracker along with the methods for retrieving its location (i.e., by means of the eNodeB position or GPS). In Section 5 field tests are presented while the relative results are reported and discussed in Section 6. Eventually, Section 7 highlights conclusions and final remarks.

## 2. Related Works

The vast diffusion of networked devices within the IoT framework occurred in the last ten years has paved the way to the emergence of a wide range of localization and tracking algorithms and techniques, with the aim to replace, or simply support GPS, in order to reduce power consumption. It is indeed well known that the acquisition of the position by means of GPS may be a time and power consuming activity: thus, GPS-based trackers are in general characterized by relatively short lifetimes, requiring frequent battery recharge or replacement.

With the aim of satisfying this requirement, localization and tracking solutions have been proposed adopting all the state-of-the-art data transmission technologies, using alternatively RSSI [1], ToA [2], TDoA [3] or AoA [4] techniques to improve the accuracy maintaining a limited power consumption. If taking into account common IoT transmission technologies, the achievable results are obviously related to the transmission ranges: in this sense, local area technologies like WiFi [5], ZigBee [6] or Bluetooth [7] have been extensively applied to indoor localization and tracking, where GPS signal is not available. Initially, indoor localization was carried out by resorting to standard RSSI trilateration algorithms. However, such techniques are prone to a reduction of accuracy whenever the environment is subject to variations, as it usually happens within indoor scenarios. To this end, [8] proposed an adaptive wireless indoor localization system aiming at overcoming the aforementioned drawback by making use of an automated database updating process along with a new fingerprinting algorithm. The database is dynamically populated by resorting to a self-locating mobile robot patrolling the environment. It regularly collects RSSIs within the localization space, and then such data is stored within the database. Similarly, indoor environments are intrinsically characterized by rooms, obstacles and walls which cause non-line-of-sight (NLoS) links hindering transmissions. This effect was alleviated by the study proposed in [9] where an identification and mitigation method for indoor and NLoS localization was devised. Indeed, it is a less environment-dependent and a priori knowledge-independent procedure turning out to be less incline to errors. However, for what concerns indoor localization, it is worth making reference to an extremely well drawn up literature survey [10] pointing out several techniques and technologies, along with a focus on localization in IoT contexts and an analysis of sundry application frameworks and benchmark performances. Conversely, such systems can be hardly employed for outdoor asset tracking due to their limited transmission ranges (in general lower than 100 m) [11]: their usage becomes totally unfeasible for long distance asset tracking, which is the application context of supply chain, where goods are shipped across whole countries, continents or even the entire world.

Long range transmission technologies are thus, by-far, more suitable for asset tracking purposes, allowing the actual position detection of goods across large areas. Traditional cellular technologies like General Packets Radio Service (GPRS) [12] or LTE [13] are obviously perfect candidates for GPS-less asset tracking: coverage is globally provided and item position can be retrieved by means of cell trilateration. This solution suffers however for high power consumption: while it can be easily applied for the acquisition of people position, thanks to mobile phones, it is not ideal for items tracking since long term functioning cannot be ensured. Since high power consumption is a critical factor for almost the most part of Machine-to-Machine (M2M) applications, a number of alternative technologies has emerged in the last ten years, leading to the definition of the already introduced LPWAN paradigm. In this context, a clear dichotomy has risen among unlicensed and licensed technologies: while the former ones include a wide range of different solutions including LoRaWAN and Sigfox, the latter ones encompass all the cellular systems.

Regarding unlicensed technologies, both LoRaWAN and Sigfox have been tested for asset tracking purposes, with different feedbacks though. Indeed, LoRa [14] and LoRaWAN [15] proved to be viable solutions to achieve decent tracking: however, the limitation concerning the use of this technology comes from the requirement of having a distributed network infrastructure, whose capillarity has to be the largest possible to localize assets also in remote areas. For this reason, solutions available in literature only focus on asset tracking in limited areas, while the lack of a global infrastructure remains the biggest limit to a large scale use for tracking purposes within supply chain context. Conversely, Sigfox was especially designed having global asset tracking in mind: indeed, such technology was patented by the eponymous French company which contextually set up a global network infrastructure whose coverage is now available in around 80 countries [16] and was provided with an ad-hoc Sigfox geo-location service called Atlas [17]. Due to these features, several works in literature proposed methodologies and systems for Sigfox-based asset tracking [18,19,20]. However, this technology too has its drawbacks: indeed, Sigfox still foresees a subscription fee, while limitations are also posed on the maximum number of packets that can be broadcast each day. These limitations are preventing for its large scale usage.

Licensed LPWANs may represent a good trade-off between the two requirements of low power consumption and capillary network coverage [21]: indeed, such technologies are especially designed for M2M communications, which have power consumption reduction as a primary target. At the same time, they rely on the cellular network infrastructure, which ensures a diffused network coverage almost worldwide. Primary leading technologies in the licensed LPWAN domain are currently LTE-M [22], also known as (LTE-MTC [Machine Type Communication]), and NB-IoT [23]: while both technologies are shaped on the M2M domain, LTE-M is characterized by higher data rates and then higher bandwidth requirements. In turn, power consumption is notably higher: for this reason, NB-IoT is the principal choice for low power applications and then for possible low power asset tracking and localization. Moreover, NB-IoT proved to be a reliable enabling technology to be employed in critical environments [24], such as whenever the transmitter is housed within metallic enclosures like it usually happens during asset tracking.

However, a relatively low number of works were published dealing with localization and positioning by means of NB-IoT: in particular, a specific attention was devoted to a novel technique introduced within the LTE framework, called Observed Time Difference of Arrival (OTDoA) [25]. This algorithm, which is based on the time difference between signals exploiting it for multilateration, was also applied for NB-IoT systems [26,27,28,29]: while [26,27,28] tested the effectiveness of this technique by means of numerical simulations, [29] performed laboratory measurements showing accuracy errors spanning within the range 50 m ÷ 70 m making the localization relatively feasible even in the circumstances of high noise environments. Other techniques that were applied for position retrieval with NB-IoT systems include Channel State Information (CSI) [30] and Received Signal Strength (RSS) [31]: however, CSI is only used for indoor localization, which is a totally different application domain with respect to asset tracking. Conversely, RSS is employed for outdoor localization and tracking: Janssen et al. [31] demonstrated the feasibility of single-cell based tracking within a urban area, achieving an error of some hundreds of meters. Such value is compliant with the accuracy requirements of asset tracking when moving towards the destination. However, when approaching the arrival point, a higher degree of precision may be required, suggesting the use of a more accurate technique.

Specifically concerning asset tracking, only two works were identified in literature [32,33]. Suryani et al. [32] only focus on the use of NB-IoT for data transmission, leaving the task of position acquisition to GPS, thus applying an approach that is partially recovered in this paper: moreover, a power consumption analysis is carried out, comparing the NB-IoT performances with the ones of traditional GPRS connectivity. Conversely, Kavuri et al. [33] suggest the usage of NB-IoT to track containers transported on cargo ships. While solutions based on the use of Unmanned Aerial Vehicles (UAV) and relay ships are presented, the paper also proposes the adoption of eNodeBs. However, only results from numeric simulations are provided, without implementing and testing on field the actual performances of a tracker.

However, both the approaches of the two previously described papers are interesting from the point of view of this paper, which combines the two techniques proposing different algorithms in order to reduce power consumption. Moreover, this paper presents the results of real field tracking activities, thus demonstrating the actual feasibility of the proposed solution: as far as the authors are concerned, no work has ever presented the field operation of NB-IoT-based localization systems which are able to perform asset tracking for long distance shipments.

## 3. System Architecture and Working Principle

### 3.1. System Architecture Description

**Figure 1:**
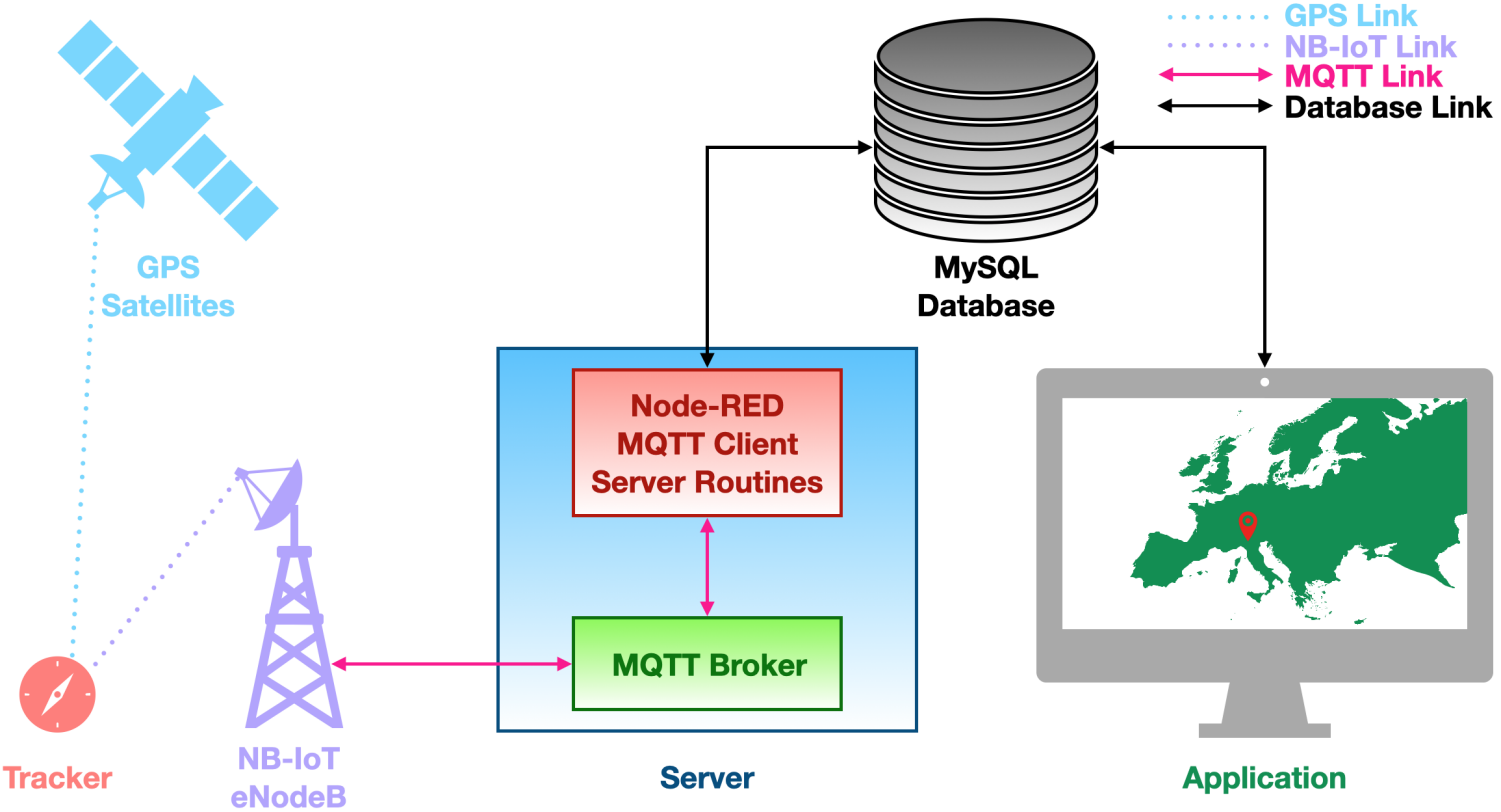
System architecture block diagram.

System architecture is outlined in Figure 1: its principal component is the tracker prototype that will be thouroughly described later on (see Section 4). Briefly, it is a device which embeds a transceiver supporting both GPS and NB-IoT technologies. The former is only used to retrieve tracker position with a sporadic regularity since such procedure entails a pretty high power consumption, while the latter is harnessed for both localisation and position broadcasting. In particular, localization via NB-IoT is just a coarse but parsimonious method because the tracker position is assumed to be the one of the relative eNodeB the tracker pairs to during network synchronisation phases. On the other hand, fetching position from GPS is by far finer though more energy requiring, and that is the reason why such strategy is not continuously put into effect. Finally, the position is sent to a remote server, by means of MQTT protocol over NB-IoT links, regardless of the method to retrieve it: in particular, when eNodeBs are employed, their identification parameters are remotely broadcast so to retrieve the relative geographic position, while when GPS is exploited, the position of the tracker is directly transmitted.

**Figure 2:**
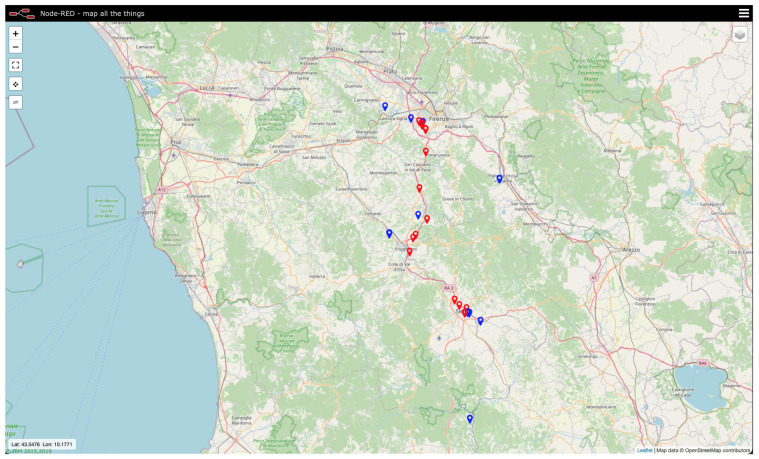
Snapshot of the tracking system web application showing the latest positions recorded by the device during its early testing stages: blue markers stand for locations coming from eNodeBs, while red markers stand for locations coming from GPS.

The eNodeB forwards the aforesaid information, by means of the MQTT protocol, to a remote server, which is composed of an MQTT broker and a Node-RED flux running an MQTT client, that is connected to the aforementioned MQTT broker, and implementing all the needed routines to manage incoming data. Node-RED [34] is a development environment, originally devised by IBM, which runs on Node.js [35]: it is grounded on a flow programming language whose building blocks are written in JavaScript. In so doing, a run-time environment executing JavaScript codes outside a browser, so to enable their employment for scripting on server side, is implemented. The MQTT client is subscribed to the same MQTT topic the MQTT client running on the tracker posts data on. Such data are then handled by the Node-RED flux and finally stored in a MySQL database. The application is implemented in Node-RED too and it can be consulted from whichever device allowing for a web browser (e.g., computers, tablets, smartphones, etc.). It retrieves tracker positions from the database and then it displays them on a map by means of markers having different colours (see Figure 2): blue for locations coming from eNodeB positions and red for the ones coming from GPS.

### 3.2. Working Modes Description

The tracker prototype is designed so to implement two alternative working principles: Mode #1 and Mode #2.

**Figure 3:**
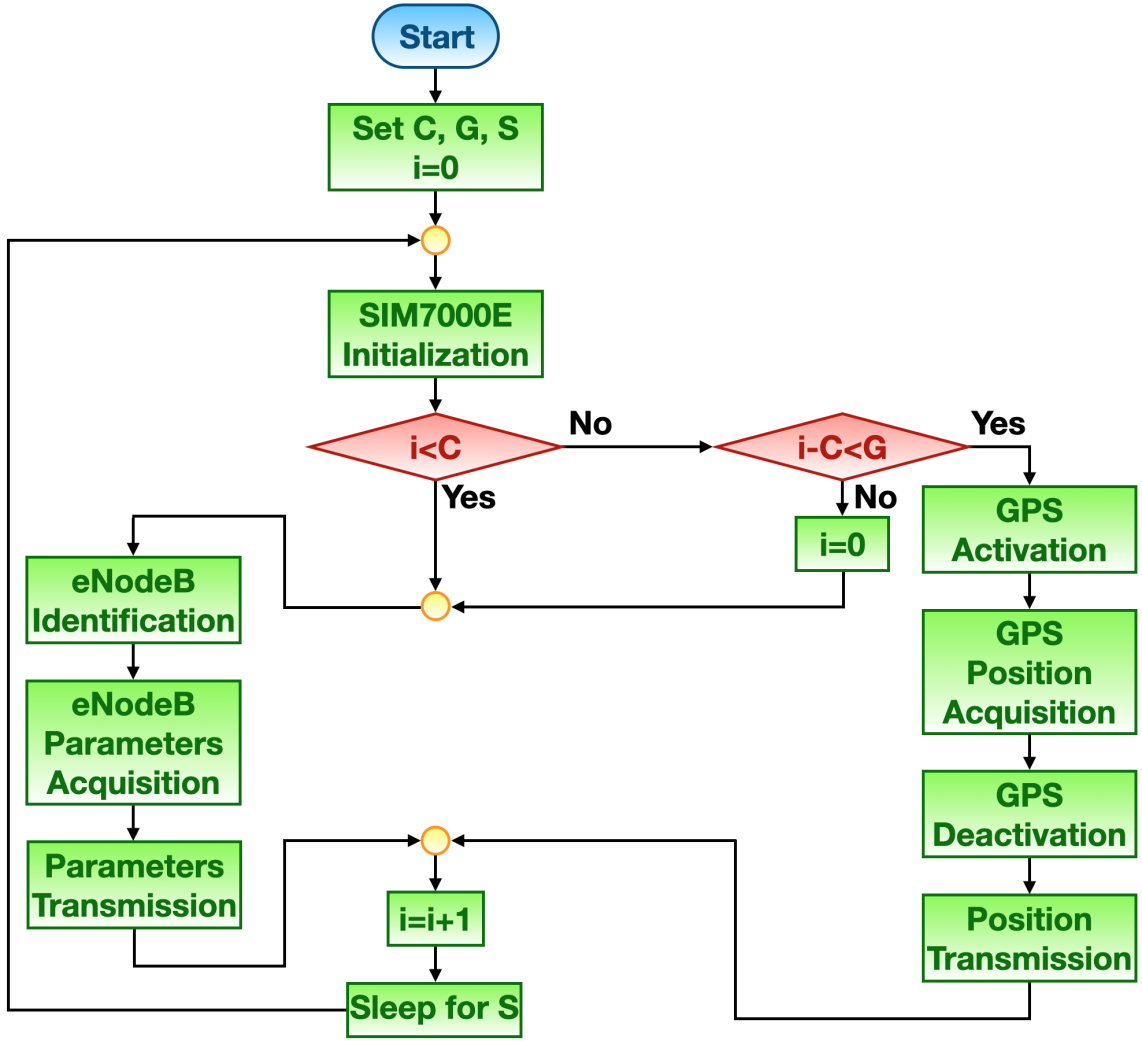
Mode #1 flow chart.

Mode #1 flow chart is depicted in Figure 3. This working principle is primarily distinguished from the interchanging of eNodeB and GPS techniques for retrieving tracker position. Let *C* be the number of consecutive tracker location acquisitions and transmissions coming from the eNodeB geographic position. On the other hand, let *G* be the number of consecutive tracker location acquisitions and transmissions retrieved from the GPS. Finally, let *S* be the amount of time the tracker spends in sleep mode after each transmission and *i* be an auxiliary counter incrementing after each transmission that is initially set equal to zero. At first, the NB-IoT module is initialized so that it can accomplish all the needed routines to synchronize to the network. Suppose i<C then the geographic position of the eNodeB the module paired to during its initialization is exploited as tracker position. Therefore, such eNodeB is identified by retrieving its distinctive parameters that are then sent via MQTT over NB-IoT to the remote server. Subsequently, the server gets eNodeB geographic position through the aforesaid parameters by making use of an online database that will be described later on. At this stage, the counter *i* is incremented and the tracker enters in sleep mode for the time *S* so to save energy. Once *S* is elapsed, the NB-IoT module repeats initialization phase. In so doing, *C* consecutive position acquisitions via eNodeB are made. Then, supposing i≥C and such that i−C<G meaning that the location coming from the GPS is used as tracker position. Thus, the GPS receivers is turned on and the tracker position is acquired. Afterwards, the GPS receiver is shut down so to save power. The acquired position is transmitted as when it is obtained from eNodeB. Subsequently, the tracker enters in sleep mode for the time *S* in order to restart its routines. Finally, suppose i≥C and such that i−C≥G thus implying that *G* consecutive position acquisitions via GPS were carried out: the counter *i* is reset and eNodeB method for retrieving tracker position is put into effect. The relative procedures resulting in position acquiring via both eNodeBs and GPS will be explained later on in Section 4.2. Of course, in order to limit power consumption, the relationship C>G should hold.

**Figure 4:**
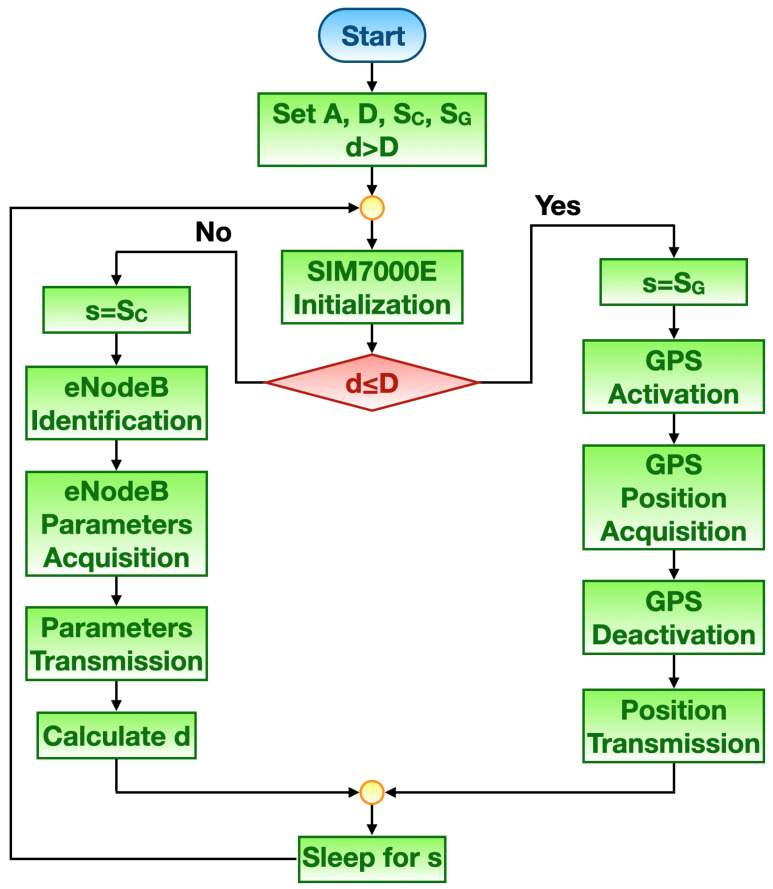
Mode #2 flow chart.

Mode #2 flow chart is reported in Figure 4. This working principle is grounded on the distance between the tracker position and the shipment arriving point. Let *A* be the position of the shipment arriving point, *D* the threshold which discerns when exploiting eNodeBs or GPS for retrieving tracker position (which directly translates into the radius of a circle around A, and having the latter as centre), and *d* the distance at hand between the tracker and the shipment arriving point. For the sake of algorithm starting, *d* is initialized as greater than *D*. Finally, let $S_{C}$ and $S_{G}$ be the amount of time the tracker spends in sleep mode respectively after eNodeBs and GPS are used for the localization, while *s* an auxiliary variable which represents the sleep time during the algorithm cycle at hand. Likewise Mode #1, at first the NB-IoT module executes initialization routines. Since d≤D, eNodeBs are employed for retrieving tracker position, thus *s* is set to $S_{C}$ and tracker position resorting to eNodeB geographic location is carried out in the same manner as for Mode #1. Then, *d* is calculated by firstly requesting via MQTT to the remote server the last acquired position. Such information is provided by the server to the tracker by means of MQTT. Then, the tracker computes *d*. Once transmission is completed, the tracker enters in sleep mode for the time *s*. As soon as d>D, the tracker got closer to *A* thus it is worthy to execute a finer tracking, hence only resorting to GPS. Therefore, *s* is set to $S_{G}$ and tracker position through GPS is accomplished in the same way as for Mode #1. Of course, in order to limit power consumption, the relationship $S_{G}>S_{C}$ should hold.

## 4. Tracker Prototype Description

### 4.1. Hardware Setup

**Figure 5:**
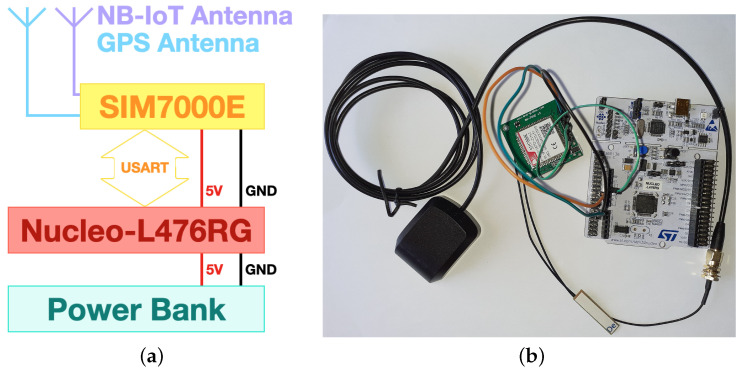
Tracker prototype: (**a**) block diagram; (**b**) hardware implementation without the power bank.

The tracker prototype block diagram is presented in Figure 5a, while its hardware implementation is shown in Figure 5b. Albeit it is still at its prototypical stage, and as it will be demonstrated later on (see Section 5), it is already able to completely fulfill its tasks. Future redesign phases will account for the reduction of hardware components, limiting them to the strictly needed ones so to further contain power consumption. The tracker core is a Nucleo-L476RG board produced by STMicroelectronics: it is a general purpose development board embedding an STM32L476 microcontroller [36]. The latter drives an NB-IoT transceiver (i.e., the SIM7000E manufactured by SIMCom [37]) via the Universal Serial Asynchronous Receiver Transmitter (USART) interface. The Nucleo board is powered via a power bank, while the communication module is powered by the Nucleo board itself.

SIM7000E is an LTE module, accounting for both LTE Machine Type Communication (LTE-MTC) and NB-IoT, which is driven via AT commands by the microcontroller. The module is designed for applications needing low latency, medium throughput, data communication in the most diverse radio propagation conditions. In addition, since the module also embeds a GPS receiver, it perfectly suits to the purpose of remote long term asset tracking. Connectivity is provided by means of a micro Subscriber Identity Module (SIM) card, belonging to the Italian telco provider TIM, which is especially devoted to M2M cellular communication scopes. Finally, the module makes use of two antennas which respectively are devoted to NB-IoT communication and to GPS signal reception.

### 4.2. Position Retrieval Methods

As it was previously hinted, tracker location may be retrieved by following two alternative procedures: assuming the position of the eNodeB the tracker pairs to when it synchronizes to the NB-IoT network as the tracker location, or by GPS.

Retrieving tracker position by exploiting the one of the eNodeB is the most parsimonious method from the point of view of power consumption since the GPS module is turned off. The drawback, though, is that this approach is quite coarse. However, supposing to track an object traveling for several hundreds of kms, the entailed error could be considered as negligible. Firstly, some of the user equipment system information must be fetched, and in particular the one identifying the eNodeB the module paired to. In particular, such information is obtained via an AT command whose response is composed of several fields. Four of the latter ones uniquely distinguish eNodeBs: the Mobile Country Code (MCC), the Mobile Network Code (MNC), the Tracing Area Code (TAC) and the Physical Cell Identifier (PCellID). Secondly, such information is conveyed to the remote server via MQTT. The latter obtains eNodeB position by resorting to an Application Program Interface (API) requiring the aforementioned data as input. The API is made available by OpenCellID service [38]: it is the largest open database in the world thus becoming a collaborative community project collecting worldwide positions of cell towers, within which eNodeBs are included, and their corresponding geographic coordinates. The aforesaid API is executed by the server by setting up an Hypertext Transfer Protocol (HTTP) request whose response includes the latitude and the longitude of the eNodeB at hand. As it was stated earlier on, retrieving the position via eNodeB is a proximity approach only. Be that as it may, it was adopted since it is the one entailing the lowest power consumption. Indeed, RSSI- and TDoA-based techniques, for instance, would require trilateration algorithms to be performed. This implicitly implies an extension of the time period during which the tracker prototype necessitates to be active thus translating into more needed energy, even in the case trilateration would be accomplished on the system server side. Therefore, a finer level of accuracy was gave up whenever eNodeBs are exploited since energy saving is concerned. Nevertheless, such drawback does not significantly hinder the overall system performances (as it can be claimed by field tests results in Section 6.2) due to the fact that in asset tracking contexts the tracker position precise knowledge is not essential, while having the flavour of how long it is until arriving point is reached in a quasi-real time fashion is much more important.

Retrieving tracker position by means of the GPS module the SIM7000E is equipped with entails a small error but it turned out to be quite energy consuming due to the activation of the GPS module. Therefore, such method is only fitfully put into effect so to extend the tracker battery lifetime. On the other hand, it is also the most immediate and easy to carry out. Indeed, at first the GPS receiver is turned on, then it seeks for enough satellites to compute its position, and when it succeeds the GPS receiver is switched off so to save energy.

## 5. Field Tests

The tracker prototype underwent two types of tests: the former ones focused on the assessment of SIM7000E power consumption during each of the working phases accounted within the aforementioned working modes; the latter ones aimed at evaluating the device tracking capabilities through two 75 km trips which were travelled several times in order to test both the working modes.

### 5.1. Consumption Tests

Since one of the main objectives of the proposed system was the reduction of the power consumption, thus aiming at prolonging the device battery lifetime, a set of measurements was performed concerning the current absorption of the system during the different phases of the position acquisition and data transmission. In order to carry out accurate measurements, current absorptions of the single phases were measured in laboratory: these values allow then to calculate the overall power absorption and possibly estimate the device lifetime according to the capacity of the chosen power source. Moreover, in the final configuration of the system just a low power microcontroller is expected to be used, rather than the entire general purpose board on which it is currently embedded. According to its datasheet, such microcontroller (i.e., the STM32L746) has current absorptions in the order of less than 1 mA in active mode and some tens of nA in shutdown mode: only the power consumption of the SIM7000E module during the different phases was then evaluated. However, power consumption between one position acquisition and the subsequent one can be assumed as negligible since the only component which has always to be turned on is the microcontroller, but in shutdown mode though.

In order to measure the power consumption of the SIM7000E, a USB-6003 NI Data Acquisition (DAQ) board by National Instruments (NI) was employed. Since the supply voltage of the module is constant and equal to V=5V, in order to calculate the power consumption only the absorbed current i(t) was acquired. The measurement was performed by putting a low resistance value resistor in series with the module: in particular, a R=1.2Ω resistor with 1% tolerance was chosen in order to minimize the loading effect while keeping an adequate level of accuracy. The voltage drop on the resistor v(t) was then acquired with the DAQ by means of LabVIEW, assuming a sampling frequency of 100 Hz. Such value was then used to calculate i(t) from Ohm’s Law:

(1) $$i\left(t\right)=\frac{v(t)}{R}.$$

While the different phases account for different timings, data acquisition was performed synchronizing its start and end with the beginning and finish time instants, respectively $t_{b}$ and $t_{f}$, of the different phases by means of code breakpoints. Then, energy consumption during the different phases was evaluated:

(2) $$E=V\sum_{t=t_{b}}^{t_{f}}i\left(t\right).$$

### 5.2. Tracking Tests

**Figure 6:**
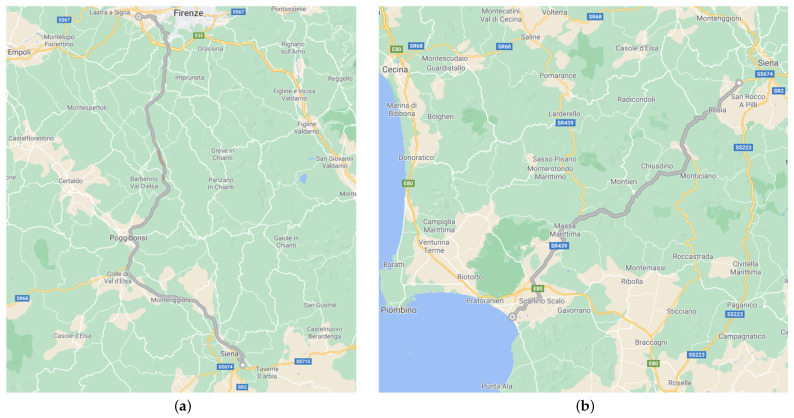
Exploited itineraries for tracking tests: (**a**) itinerary including dirt roads, urban streets and freeways; (**b**) itinerary including urban streets, suburban streets and wooden roads.

During these trials, the tracker prototype was carried around two 75-km long trips (see Figure 6), and both of them were accomplished by car within which the tracker prototype was placed. In order to test both working modes, such trips were travelled back and forth. Each leg of the former trip averagely took 65 min, while each leg of the latter one averagely took 80 min. In particular, both working modes were tested for a round trip by choosing appropriate relative parameters (see Section 3.2) which were aimed at stressing the device: as it will be seen later on, those parameters could have been relaxed, especially for limiting power consumption. However, this strategy was not put into effect, thus obtaining a position oversampling, so to test the overall system effectiveness, along with the fact that no power supply issues could arise since the tracking prototype was powered via a 10,000 mAh power bank. The first itinerary (see Figure 6a) was composed of sundry street typologies: dirt roads (where the mean car speed was of 15 km/h), urban streets (where the mean car speed was of 45 km/h) and freeways (where the mean car speed was of 90 km/h). Moreover, in some road stretch traffic jams occurred, while in other ones flowing traffic was present. In addition, and especially within the freeways, tunnels were passed through which forbade data transmission. The second itinerary (see Figure 6b) was composed of sundry street typologies too: urban streets (where the mean car speed was of 45 km/h), suburban roads (where the mean car speed was of 70 km/h) and wooded roads (where the mean car speed was of 40 km/h). This itinerary was characterised by flowing traffic. However, within the wooded roads signal coverage of both GPS and NB-IoT was terribly hindered due to the high density of tall trees. Finally, for the sake of simplicity, the tracker prototype was housed within a cardboard box by letting out only the GPS antenna so to be able to receive satellites signals.

Mode #1 (see Figure 3) was tested by setting the relative parameters as follows: C=2, G=1 and S=30 s. On the other hand, Mode #2 (see Figure 4) was tested by setting the relative parameters as follows: D=10 km, $S_{C}=30$ s and $S_{G}=10$ s.

In order to assess the location error whenever eNodeBs positions are accounted, additional tests were sorted out which took place on the same itineraries of Figure 6 that were travelled back and forth. In such circumstance, the tracker prototype functioning scheme was modified so to retrieve a pair of positions coming from both the GPS and the eNodeB. In doing so, for each measurement the error can be evaluated by calculating the distance between the eNodeB and the position resulting from the GPS. The latter can be considered as the actual position of the tracker prototype owing to the fact that the GPS module embedded within the SIM7000E features an accuracy of 2.5 m. This is also the reason why there is no point in doing such sort of tests focusing on GPS position.

## 6. Results and Discussion

### 6.1. Consumption Tests Results

**Figure 7:**
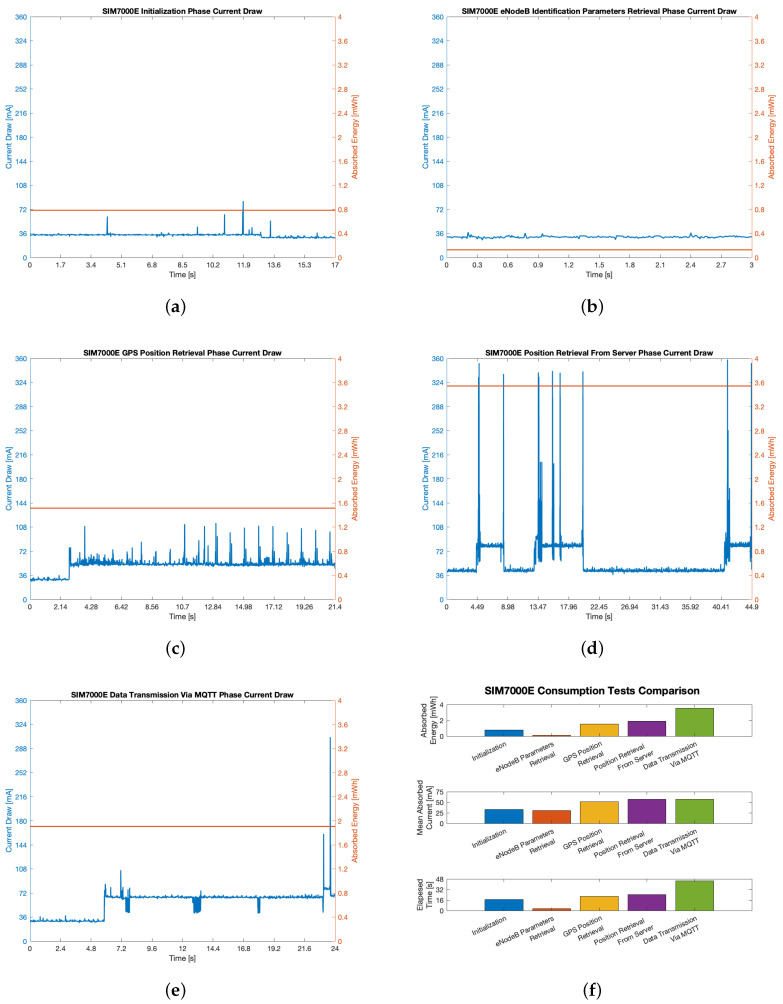
Consumption tests results: (**a**) Module intialization phase; (**b**) eNodeB identification parameters retrieval phase; (**c**) GPS position retrieval phase; (**d**) Position retrieval from server phase; (**e**) Data transmission via MQTT phase; (**f**) Comparison between the aforementioned phases in terms of absorbed energy, mean absorbed power and temporal duration.

Figure 7 shows the acquired data related to current absorption and energy consumption, calculated according to Equation (2), of the SIM7000E module during five different phases. In particular, the following five phases were taken into account for both GPS- and eNodeB-based localization methods:

1. Module initialization phase (see Figure 7a), during which the SIM7000E accomplishes its setup routines;
2. eNodeB identification parameters retrieval (see Figure 7b), during which the SIM7000E fetches the MCC, MNC, TAC and PCellID of the eNodeB it paired to;
3. GPS position retrieval (see Figure 7c), during which the embedded GPS receiver is queried for the tracker position;
4. Position retrieval from server (see Figure 7d), during which the remote server forwards the last acquired position to the tracker prototype via MQTT in order to evaluate *d* whenever working Mode #2 is put into effect (see Section 3.2);
5. Data transmission via MQTT (see Figure 7e), during which the retrieved position is broadcast to the remote server regardless of the method adopted to acquire it.

Looking at the trends related to the five phases, and by looking at Figure 7f, it is possible to point out that the energy hungriest one is by far phase 5: indeed, data transmission via MQTT requires the longest time to be accomplished along with the maximum absorbed energy, thus resulting in a considerable amount of mean absorbed power. Nevertheless, any phase involving data transmission or reception via MQTT requires a considerable quantity of energy: the average current absorption for this activity is around 70 mA with peaks up to 300÷350 mA. However, such value also depends on the strength of the received signal from eNodeBs: for this reason, an absolute peak value is difficult to estimate. In comparison, position acquisition by means of GPS satellites connection requires a lower amount of energy, with an average current absorption in the order of 60 mA.

Besides analyzing current absorption trends, the duration of each phase has to be taken into account so as to estimate the actual power consumption of each position retrieval operation, either with eNodeB or GPS. Indeed, these time spans are strongly dependent on network connection availability, both with the cellular network and with GPS satellites. These temporal lengths are shown in Figure 7. They have to be assumed as examples that may undergo considerable variations according to the different sites and environmental conditions which directly affect cellular coverage as well as GPS reception. Such variations are however more consistent for the GPS position retrieval phase, which strongly depends on a fast successful connection with GPS satellites that in many cases may not be available: as a matter of fact, while in the example shown in Figure 7c this phase lasts ∼21.4 s, its duration may easily grow up to a minute or even more. Of course, once a connection is established, subsequent position retrievals may be quickly achieved. However, such operation mode would require a continuous powering of the communication module, with a huge power consumption which is in contrast with the low power requirement of the tracking system. Indeed, keeping the GPS radio module, together with the SIM7000E module, always switched on would reduce the lifetime of the device to a couple of days or even less. For this reason, duty cycling is adopted thus entailing the module restarting every time a new position is retrieved. Similar considerations apply to the other critical phase, which is data transmission via MQTT: such phase is however present for any kind of position retrieval technique and cannot be avoided regardless of the position retrieval mode.

In Figure 7a comparison related to absorbed energy (expressed in mWh), mean absorbed current and duration of each of the phases is provided. Such comparison is helpful to better understand the actual contribution of the different phases to the overall consumption of the module during its operating cycles according to the both Mode #1 and Mode #2. Indeed, besides comparing energy consumption and operation timings, a comparison related to average current absorption is also given: such information is helpful to better compare the actual consumption regardless of the duration of the single phases. This comparison shows that a limited difference is detectable for what concerns current absorption: indeed phases 1 and 2 have an average current absorption around 30 mA (respectively, 33.26 mA and 30.73 mA for phase 1 and 2), while for phases 3–5 this value grows above 50 mA (respectively, to 50.89 mA, 57.15 mA and 56.78 mA for the three phases). This result suggests the importance of identifying the operation mode which allows to reduce as much as possible the overall power consumption since not all of the phases are accounted by each of the working mode. For instance, phase 4 is not envisaged at all in Mode #1, while it may be performed a limited number of times in Mode #2 according to the relative parameters values selection.

In order to better understand the power requirement of the aforesaid working modes (i.e., Mode #1 and Mode #2), an estimation of their energy consumption considering the phases they account for can be made.

Mode #1 foresees either the position retrieval by acquiring eNodeB parameters, or the usage of GPS position. Finally, the acquired data is transmitted regardless of which technology was used for localization. These two procedures, which can be named as Procedure A and Procedure B so to ease the treatise, are in turn consecutively executed according to the value of parameters *C* and *G* (see Section 3.2): both of them require phases 1 and 5, but Procedure A additionally requires phase 2 only, while Procedure B furthermore needs just phase 3. Then, by resorting to data resulting from the consumption tests shown in Figure 7, the following energy consumption values $E_{A}$ and $E_{B}$ can be calculated for the two procedures:

(3) $$E_{A}=E_{1}+E_{2}+E_{5}≃0.79\;\mathrm{mWh}+0.13\;\mathrm{mWh}+3.54\;\mathrm{mWh}=4.46\;\mathrm{mWh},$$

(4) $$E_{B}=E_{1}+E_{3}+E_{5}≃0.79\;\mathrm{mWh}+1.51\;\mathrm{mWh}+3.54\;\mathrm{mWh}=5.84\;\mathrm{mWh},$$

where $E_{1}$ to $E_{5}$ are the energy consumptions of the respective phases. From these results it is evident that Procedure B requires around 30% more energy with respect to Procedure A, thus meeting expectations on the fact that retrieving position via GPS is heavier with respect to eNodeB from an energetic point of view. Moreover, while the timing of Procedure A can experience limited fluctuations, Procedure B time can notably increase in case of low GPS signal reception.

Conversely, Mode #2 requires an additional procedure, namely Procedure C, with respect to Mode #1: if d>D (see Section 3.2), after the transmission of eNodeB parameters, the actual tracker position has to be retrieved from the server in order to calculate *d* and then possibly switch to Procedure B if d≤D. Such operation may be either performed every time that eNodeB parameters are retrieved and transmitted or just occasionally according to a pre-defined frequency. However, the energy consumption $E_{C}$ of this procedure can be evaluated taking into account the fact that four phases are now required (i.e., phases 1, 2, 4 and 5):

(5) $$E_{C}=E_{1}+E_{2}+E_{4}+E_{5}≃0.79\;\mathrm{mWh}+0.13\;\mathrm{mWh}+1.91\;\mathrm{mWh}+3.54\;\mathrm{mWh}=6.37\;\mathrm{mWh}.$$

This procedure is the energy hungriest one, even if a consideration about the timing must be born in mind, since in this procedure elapsed time fluctuations are limited with respect to GPS position retrieval. For this reason, in case of poor GPS coverage, Procedure C may turn to be more efficient with respect to Procedure B.

In order to roughly estimate the tracker prototype lifetime, the following assumptions are made:

- The power consumption of the microcontroller that is expected to replace the Nucleo board is assumed to be negligible. As it was previously hinted (see Section 5.1), such low power microcontroller has current absorptions of 30 nA in shutdown mode and of 3.12 mA in active mode. While power consumption in shutdown mode has limited influence on power consumption, the one in active mode is at least two orders of magnitude lower than the one of SIM7000E module, and then negligible;
- A 3000 mAh capacity is assumed for the batteries employed to power the system. This value is in line with off-the-shelf AA 1.5 V alkaline batteries and almost half of the one of 3.7 V 18,650 Li-ion batteries which are commonly used in a wide range of applications requiring a relevant amount of energy. The employed batteries are expected to be placed in series in order to achieve the required voltage: for example, by making use of two Li-ion 18,650 batteries in series a nominal voltage of 7.4 V can be reached that suffices to provide the 5 V required voltage.

The average energy consumption of Mode #1 can be generally estimated as follows:

(6) $$E_{Mode\#1}=\frac{CE_{A}+GE_{B}}{C+G}.$$

However, in order to provide a numeric estimate so to be compared with the results that will be find later on, and assuming a continuous 5 V powering voltage, let C=G thus leading to

(7) $$E_{Mode\#1}=\frac{E_{A}+E_{B}}{2}=\frac{4.46\;\mathrm{mWh}+5.84\;\mathrm{mWh}}{2}=5.15\;\mathrm{mWh}=1.03\;\mathrm{mAh}$$

Assuming a battery capacity of 3000 mAh as explained above, the total number of position retrieval operations $N_{Mode\#1}$ can then be estimated:

(8) $$N_{Mode\#1}=\frac{3000\;\mathrm{mAh}}{1.03\;\mathrm{mAh}}≃2913.$$

This value can be exploited to estimate the average transmission frequency $f_{Mode\#1}$ according to the expected lifetime of the system. If, for example, the tracking activity is expected to last for one month (that can be assumed as a reasonable duration for a long term monitoring), the position retrieval rate can be calculated as follows:

(9) $$f_{Mode\#1}=\frac{2913}{1\;\mathrm{month}}=\frac{2913}{2592\cdot 10^{3}\;s}≃\frac{1}{890}\mathrm{Hz}.$$

This value roughly corresponds to one transmission every 15 min.

Moving to Mode #2, some additional considerations must be made. Indeed, in this case the tracking is grounded on two different methods (see Section 4.2): a first one, less accurate, where GPS localization is not used and only eNodeB is used; and a second one, only based GPS and therefore on Procedure B. The first method is set on the adoption of Procedure C, which is mandatory to identify the current position and calculate the distance *d* from the point of arrival, which is possibly used to switch to Procedure B. However, such method (which is put into effect given that d>D) may be accomplished by only just executing Procedure C, entailing a considerable power consumption, or by alternating Procedure C with Procedure A. Such alternation is not envisaged with Mode #2 explanation (see Section 3.2) since at first just the working mode clarification was provided and also because this interchange between Procedure A and C could be put into effect as an expedient to further reduce power consumption. In case of continuous Procedure C execution, the average energy consumption $E_{Mode\#2_{C}}$ is obviously equal to $E_{Mode\#2_{C}}=E_{C}=6.37$ mWh ≃ 1.27 mAh considering a continuous 5 V powering voltage, while in case of alternation, assuming for example one Procedure C every ten position retrievals, it can be estimated as follows:

(10) $$E_{Mode\#2_{N_{Mode\#2_{A⟷C}}}}=\frac{9E_{A}+E_{C}}{10}=\frac{40.14\;\mathrm{mWh}+6.37\;\mathrm{mWh}}{10}≃4.65\;\mathrm{mWh}=0.93\;\mathrm{mAh}.$$

Since in Mode #2 the tracker prototype is expected to spend more time in the condition d>D, the relative lifetime estimate and number of retrieved positions are evaluated in a worst case scenario, thus only considering the case in which d>D by accounting for both continuous Procedure C and for the alternation of Procedures A and C. Hence, the total number of position retrieval operations for the aforesaid cases are in turn $N_{Mode\#2_{C}}$ and $N_{Mode\#2_{A⟷C}}$:

(11) $$N_{Mode\#2_{C}}=\frac{3000\;\mathrm{mAh}}{1.27\;\mathrm{mAh}}≃2362,$$

(12) $$N_{Mode\#2_{N_{Mode\#2_{A⟷C}}}}=\frac{3000\;\mathrm{mAh}}{0.93\;\mathrm{mAh}}≃3226.$$

With these values, the average transmission frequency for a one month tracking activity can the be calculated as:

(13) $$f_{Mode\#2_{C}}=\frac{2362}{1\;\mathrm{month}}=\frac{2362}{2592\cdot 10^{3}\;s}≃\frac{1}{1097}\mathrm{Hz},$$

(14) $$f_{Mode\#2_{N_{Mode\#2_{A⟷C}}}}=\frac{3226}{1\;\mathrm{month}}=\frac{3226}{2592\cdot 10^{3}\;s}∼\frac{1}{803}\mathrm{Hz},$$

which roughly correspond to one transmission every 18 min and every 13 min respectively.

According to this analysis, energy requirements of both the working modes (i.e., Mode #1 and Mode #2) are comparable. However, this is a worst case analysis and still both the techniques potentially allow for a month-term tracking by acquiring more than a position per hour. Therefore, such performances can be considered as satisfactory. However, both the working modes can be further efficient by thoroughly setting the relative parameters aiming at reaching a trade-off. In Mode #1, for instance, setting C≫G, along with choosing *S* adequately high translates into a massive reduction of power consumption. This result is due to both Equation (6) and to the fact that $E_{A}<E_{B}$. In addition, augmenting *S* implies the microcontroller to spend more time in sleep mode. Whereas, in Mode #2, better performances from the point of view of energy requirements can be achieved by augmenting $S_{C}$ and $S_{G}$ (for the same reasons given above concerning the time the microcontroller spends in sleep mode), as well as by setting $S_{C}\gg S_{G}$. Finally, as it was previously hinted, adopting an alternation between Procedures A and C whenever d>D entails a valuable power saving.

### 6.2. Tracking Tests Results

Both the working modes turned out to be successful from the point of view of the tracking purposes, albeit they differently performed, achieving a quasi-real time latency. However, the major drawback of the overall system has to be underlined. Indeed, GPS receivers need to be placed in such a way to have a clear view of the sky so to correctly receive satellites signals (as it was made during tests of Section 5.2). On the contrary, LTE antenna does not need to satisfy this constraint in order to ensure NB-IoT links. To this end, Mode #2 would guarantee better performances whenever the tracker will be employed for shipping in which the tracker installing methods are not known a priori. Indeed, the aforesaid working principle makes use of the GPS only when the tracker is arbitrarily close to the arriving point. However, this implicitly implies that the requested granularity for the tracking has to be relatively poor.

Tracking tests results will be reported in detail below. In particular, they will be shown on a map where blue dots stand for locations coming from eNodeBs, while red squares stand for locations coming from GPS. Concerning the former ones, they could seem to be fairly few albeit it is not so: whenever the tracker prototype retrieves its position through the one of the eNodeB it paired to, it is highly possible that an eNodeB is exploited more than once. This phenomenon entails many overlapping blue dots on the map which resemble as they were only one. On the other hand, this could also occur for positions coming from GPS, even though it is far more unlikely since it translates into the fact that the tracker stood still.

#### 6.2.1. Mode #1

**Figure 8:**
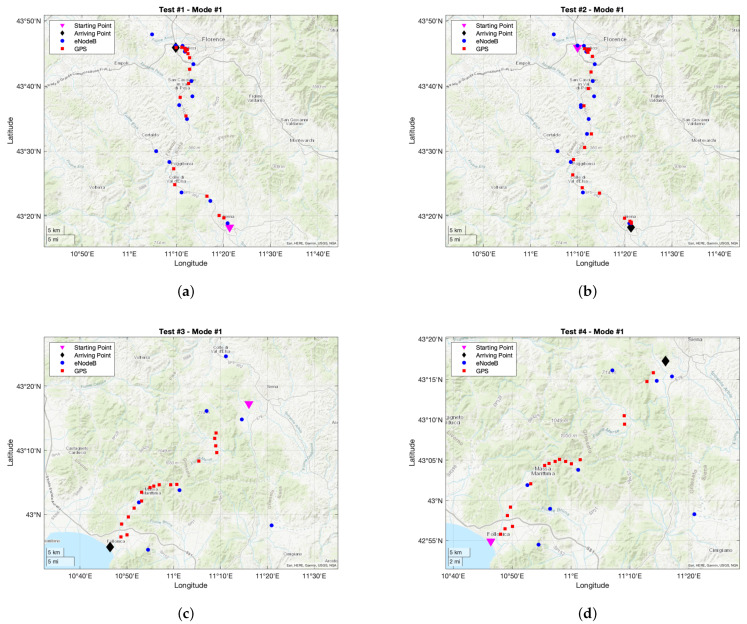
Tracking tests results exploiting working Mode #1: (**a**) outward trip of itinerary of Figure 6a; (**b**) return trip of itinerary of Figure 6a; (**c**) outward trip of itinerary of Figure 6b; (**d**) return trip of itinerary of Figure 6b.

Tracking tests results based on working Mode #1 are reported in Figure 8 for both the legs of the round trip and for both the trips of Figure 6. For what concerns the itinerary of Figure 6a, 54 positions were retrieved during the outward (i.e., 37 coming from eNodeBs and 17 coming from GPS), and 53 for the return (i.e., 32 coming from eNodeB and 21 coming from GPS). At first sight, it could seem that too few positions were obtained provided that parameter *S* was set to 30 s. However, such result is far from being unexpected since SIM7000E initialization phase has a highly variable temporal duration which unpredictably reduces the time basis on which the position is sampled. Similarly, even the other phases have a varying duration which is inversely proportional to the quality of signal coverage for both NB-IoT and GPS. Indeed, *S* must be always considered as a position retrieval time basis lower bound. Conversely, an anomalous outcome is that the number of locations deriving from eNodeBs should be twice the ones coming from GPS since $S_{C}=2$ and $S_{G}=1$, while the actual ratio approximately is 2.17 for the outward and 1.52 for the return. The reason why it happened is that the firmware running on the microcontroller deals with possible abnormal behaviours, stemming from hardware or software faults, by promptly acting with a firmware reset thus preventing blocks which would cause an indefinite deadlock, though it also implies the loss of the retrieved position at hand. Such faults may be due to sundry sources like the inability to accomplish the initialization phase because of network errors or lack of coverage.

Conversely, for what regards the itinerary of Figure 6b, 52 positions were retrieved during the outward (i.e., 35 coming from eNodeBs and 17 coming from GPS), and 51 for the return (i.e., 34 coming from eNodeBs and 17 coming from GPS). Similar conclusions can be drawn with respect to the ones of the other itinerary, and they result from the same assumptions and considerations. With respect to the ratio of location sources, it is approximately 2.06 for the outward and 2 for the return. In addition, Figure 8c,d clearly show the lack of coverage occurring at nearly half of the trip due to the wooden roads: in such spots barely no GPS locations were fetched.

In light of this, and in spite of the just mentioned shortcomings, Mode #1 proved to be highly precise thanks to the fact GPS is queried a conspicuous amount of times, as it can been seen by comparing the sub-figures Figure 6 with the ones of Figure 8.

#### 6.2.2. Mode #2

**Figure 9:**
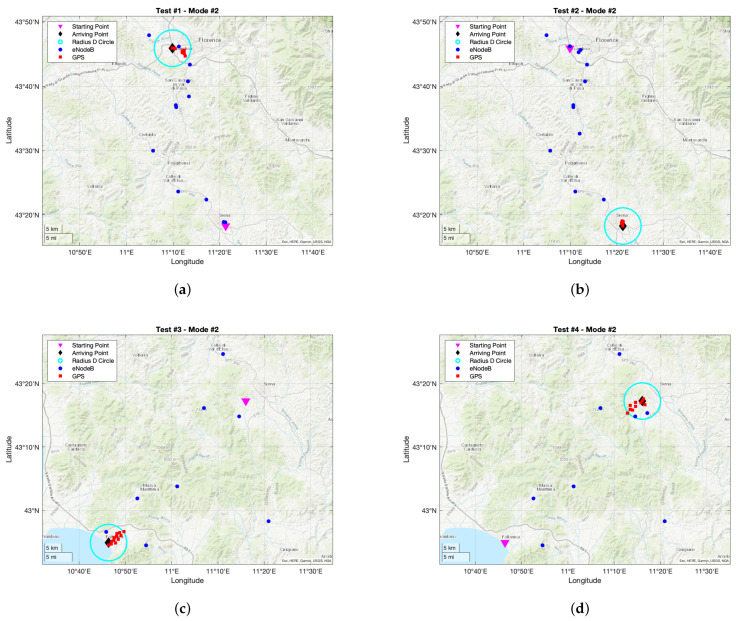
Tracking tests results exploiting working Mode #2: (**a**) outward trip of itinerary of Figure 6a; (**b**) return trip of itinerary of Figure 6a; (**c**) outward trip of itinerary of Figure 6b; (**d**) return trip of itinerary of Figure 6b.

Tracking tests results based on working Mode #2 are reported in Figure 9 for both the legs of the round trip and for both the trips of Figure 6. For what concerns the itinerary of Figure 6a, 42 positions were retrieved during the outward (i.e., 23 coming from eNodeBs and 19 coming from GPS), and 44 for the return (i.e., 26 coming from eNodeB and 18 coming from GPS). Similarly as what happened during Mode #1 tests, a minor number of positions was experienced with respect to the expected one (since parameters $S_{C}$ and $S_{G}$ were respectively set to 30 s and 10 s) for the same reasons concerning both firmware automatic resetting due to hardware and software faults, and variable temporal duration of each phase the SIM7000E accomplishes during functioning (e.g., initialization, MQTT transmission). These shortcomings were previously introduced in detail (see Section 6.2.1). In addition, and as the working principle prescribes, GPS was never exploited whenever the tracker was outside the circle having radius *D* thus entailing a notable power saving. It must underlined that eNodeB positions within such circles were only retrieved when the tracker was farther than *D* from the arriving point.

On the other hand, for what concerns the itinerary of Figure 6b, 38 positions were retrieved during the outward (i.e., 26 coming from eNodeBs and 12 coming from GPS), and 33 for the return (i.e., 19 coming from eNodeBs and 14 coming from GPS). Once again, similar conclusions can be formulated with respect to the ones of the other itinerary, and of course they emerge form the same assumptions and considerations.

This working mode is by far coarser than the other one (as it can be deduced by making a comparison amid sub-figures of Figure 6 with the ones of Figure 9), nevertheless it is the one entailing the minor power consumption. However, as it was previously hinted, this working mode is the most robust one whenever the tracker GPS antenna cannot be always placed in spots where a clear view of the sky is ensured.

#### 6.2.3. Tracking Error Test

**Figure 10:**
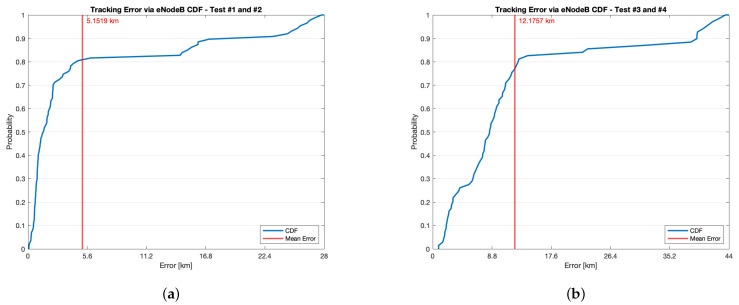
Tracking accuracy test results concerning tracking error CDF and mean value for (**a**) the itinerary of Figure 6a and for (**b**) the itinerary of Figure 6b.

Tracking error test results are shown in Figure 10 where the Cumulative Distribution Function (CDF) of the error, along with the relative mean value are plotted. During these tests similar shortcoming with respect to the ones occurred during the tracking trials were experienced. They were due to the fact that both the itineraries are composed by sundry street typologies including tunnels and tall trees woods that obviously interdicted communications relying on both GPS and NB-IoT. Concerning the tests taking place in the trip of Figure 6a, 87 samples were collected thus resulting in as much tracking error measurements. However, this do not prevent from getting a flavour of the system tracking accuracy. The mean tracking error was of 5.1519 km. Such value in itself hints at a rather high accuracy of the system. Moreover, from the CDF it can be stated that the tracking error is under the mean value with a probability of 0.81. Similarly, with a probability respectively of 0.25, 0.5 and 0.75 the error is smaller than 0.76 km, 1.5 km, and 3.84 km. However, bigger tracking errors were also experienced: in particular, an error in turn greater than 15 km and 23 km was measured with a probability of 0.15 and 0.1, while no errors greater than 28 km were sampled.

Conversely, during the tests carried out in the trip of Figure 6b, 69 samples were collected which brought as much tracking error measurements. The mean tracking error was of 12.1757 km. Such value was higher than the one of the other test, mainly due to the fact that fewer eNodeBs are installed throughout the itinerary with respect to the other trial. This fact is also underlined by comparing the results in sub-figures of Figure 8 and of Figure 9. In addition, from the CDF it can be claimed that the tracking error is under the mean value with a probability of 0.77. Likewise, with probability respectively of 0.25, 0.5 and 0.75 the error is smaller than 3.83 km, 8.46 km and 11.75 km. Nevertheless, bigger tracking errors occurred as well: specifically, an error greater than 23 km and 40 km was measured with a probability in turn of 0.15 and 0.1, whereas no errors greater than 44 km were experienced.

In sight of this, it can be claimed that the system features a good level of accuracy for asset tracking purposes. Finally, it should be underlined the fact that the tracking error, and in turn the system accuracy, is strongly dependent on the eNodeB installation sites. Indeed, despite the two tested itineraries were of the same length, they are located in geographic areas that does not share the same density of eNodeBs, resulting in different tracking error statistics. Obviously, eNodeB locations are defined a priori and the tracking system can never have any effect on them.

## 7. Conclusions

This paper aimed at showing the architecture of an innovative tracking system enabled by NB-IoT cellular technology. In particular, the system is composed of a prototype of a tracker and a back-end side which includes a dedicated server, a database and a web application from which users may check tracker location in real time. The tracker prototype embeds a transceiver module which is capable of simultaneously exploiting two technologies: GPS and NB-IoT. Such facilities are employed as follows: the former for tracking scopes, while the latter for both tracking and communication purposes. To this end, two alternative methods for position retrieval were devised: one is coarse but low power, while the other one is finer but more energy requiring. Specifically, the coarse approach envisages tracker location acquisition as the one of the eNodeB the communication module paired to during its initialization phase. Conversely, the finer one entails the use of the GPS for position retrieval. The aforementioned methods are carried out according to two working principles: Mode #1 interchanges a certain number of consecutive position retrieval by making use of eNodeBs, to a certain number of consecutive GPS-based position retrievals; Mode #2 only uses position retrieval via eNodeB apart whenever the tracker is close enough to the arriving point since solely GPS is employed.

Tests were focused on a twofold scope: power consumption and tracking capabilities. The former found out that energy requirements of both the working modes (i.e., Mode #1 and Mode #2) are comparable. Nevertheless, power consumption estimates were carried out by accounting for a worst case. Notwithstanding, both the techniques potentially allow for a month-term tracking by acquiring more than a position per hour. Therefore, such performances can be considered as satisfactory. On the other hand, tracking capabilities were assessed by testing both working principle (i.e., Mode #1 and Mode #2) through two 75-km long trips. Trial outcomes pointed out that Mode #1 is by far more precise than Mode #2, but Mode #2 is potentially more robust than the other one. Yet, the overall system effectiveness was proved despite the tracker is only at its prototypical stage. Finally, the tracking error was also tested within the two itineraries. While each of them provided its own level of accuracy, the tracking error statistics may be deemed as satisfactory for asset tracking purposes showing that the error is strongly dependent on the geographic deployment of eNodeBs.

## Data Availability

The data presented in this study are available on request from the corresponding author.
